# 

**Published:** 1866-07

**Authors:** 


### CONTENTS.

ORIGINAL COMMUNICATIONS.

Dental Materia Medica, ... 285
Orthodontia, ... 288
Great Things, ... 294
What We Most Need, ... 297
Stuffing Teeth, ... 299

PROCEEDINGS OF SOCIETIES.

Constitution and By-Laws of the Central States Dental Association, ... 301
Minutes of the Twenty-Second Annual Meeting of the Mississippi Valley Association of Dental Surgeons, ... 307
Massachusetts Dental Society, ... 315

SELECTIONS.

Medical Teaching, ... 318
Medical Education—New Professorships in the Medical Depart'nt of Harvard University 318
Another Death from Chloroform, ... 321
Chloroform, ... 322
A Human Curiosity, ... 322
St. Louis Odontological Society, ... 322
American Dental Association, ... 323

EDITORIAL.

A Bill to Regulate the Practice of Dentistry, ... 324

Obituary—D. C. Ambler, M. D., ... 328

Obituary—Dr. S. L. Hamlin, ... 385

